# Combined predictive effects of sentential and visual constraints in early audiovisual speech processing

**DOI:** 10.1038/s41598-019-44311-2

**Published:** 2019-05-27

**Authors:** Heidi Solberg Økland, Ana Todorović, Claudia S. Lüttke, James M. McQueen, Floris P. de Lange

**Affiliations:** 1 Medical Research Council Cognition and Brain Sciences Unit, University of Cambridge, Cambridge, UK; 2 Oxford Centre for Human Brain Activity, University of Oxford, Oxford, UK; 3 Donders Institute for Brain, Cognition and Behaviour, Radboud University, Nijmegen, The Netherlands; 4 Max Planck Institute for Psycholinguistics, Nijmegen, The Netherlands

**Keywords:** Neuroscience, Cortex

## Abstract

In language comprehension, a variety of contextual cues act in unison to render upcoming words more or less predictable. As a sentence unfolds, we use prior context (*sentential constraints*) to predict what the next words might be. Additionally, in a conversation, we can predict upcoming sounds through observing the mouth movements of a speaker (*visual constraints*). In electrophysiological studies, effects of visual constraints have typically been observed early in language processing, while effects of sentential constraints have typically been observed later. We hypothesized that the visual and the sentential constraints might feed into the same predictive process such that effects of sentential constraints might also be detectable early in language processing through modulations of the early effects of visual salience. We presented participants with audiovisual speech while recording their brain activity with magnetoencephalography. Participants saw videos of a person saying sentences where the last word was either sententially constrained or not, and began with a salient or non-salient mouth movement. We found that sentential constraints indeed exerted an early (N1) influence on language processing. Sentential modulations of the N1 visual predictability effect were visible in brain areas associated with semantic processing, and were differently expressed in the two hemispheres. In the left hemisphere, visual and sentential constraints jointly suppressed the auditory evoked field, while the right hemisphere was sensitive to visual constraints only in the absence of strong sentential constraints. These results suggest that sentential and visual constraints can jointly influence even very early stages of audiovisual speech comprehension.

## Introduction

If, during an English conversation, you see your friend put her upper teeth against her lower lip, you would know which kind of speech sound to expect next: a labiodental fricative consonant, i.e. either /f/ or /v/. These two different speech sounds share the same *viseme* (the facial gesture we can see when someone utters a speech sound). Visemes have been suggested to function as predictive cues to upcoming acoustic speech^1^. However, sentence context may also provide you with valuable information about upcoming speech. For example, your friend might say, “*The firewood immediately caught […]*”. Such a context enables you to draw on both your linguistic and world knowledge (e.g., about the expression “to catch fire” and about the properties of firewood). In this case you might be able to predict “caught fire” as a likely ending. Sentential constraints could thus also serve as predictive cues^2^. In the current study, we asked whether visual and sentential constraints jointly influence language comprehension and, if so, when they do so. Specifically, can contextual cues (e.g., sentential information about upcoming “fire”) modulate uptake of visual cues (e.g., mouth movements about the upcoming /f/-/v/ viseme) early in the comprehension process?

During speech recognition, visual information often precedes auditory information^3^. Visemes can communicate information about the place of articulation of a speech sound earlier and more efficiently than the acoustic signal alone^4^. While visemes can therefore be used to predict upcoming acoustic speech, the degree to which they can do so depends on the level of certainty with which they signal (classes of) speech sounds. We will refer to this as *viseme salience*. The viseme for /f/ and /v/ is highly salient, as critical aspects of the articulation of these sounds are clearly visible. Visemes such as those belonging, for example, to velar consonants (/g/ and /k/) are less salient, since the constriction that produces the sound is not visible. The available visual information associated with velar consonants is consistent with sounds produced at other posterior places of articulation. Viseme salience is reflected in an early neuronal response: the auditory N1 peaks earlier and has a lower amplitude with more salient visemes^5–7^, a phenomenon we will refer to as the *viseme effect*. Importantly, the viseme effect has been observed when single words, or even meaningless syllables are presented^8^, with no preceding sentence context.

The auditory N1 is an event-related potential (or event-related field) peaking around 100 ms following a change in the acoustic environment^9^. It is also considered to reflect early stages of language processing such as prelexical acoustic-phonetic analysis^10–12^. Although traditionally seen as a stimulus-driven response to an auditory event, the N1 has also been found to be sensitive to higher-level cognitive states like attention and expectation^9,13,14^. A possible interpretation of the viseme effect, then, is that visemes serve to influence the probability of which sounds might be encountered next, with more salient visemes enabling more accurate predictions. Many viseme studies, however, used isolated phonemes or syllables^5,7,8,15^, thus trading off tighter experimental control for slightly lower ecological validity. One study that did employ an audiovisual paradigm with speakers uttering entire sentences, interestingly, found a reverse viseme effect (a stronger N1 for high visual saliency) confined to the right hemisphere^16^.

In addition to viseme salience, there is considerable evidence to suggest that we are able to use grammatical, semantic and pragmatic sources of information in sentences to make predictions about what words will come next in those sentences^17–24^. One possibility is that sentence context, similarly, serves to influence the probability of which sounds might be encountered next, through facilitating predictions not only about the meaning of the incoming word, but also about its form. In electrophysiological studies, however, sentence context predictions have been shown primarily to modulate the neural response in the N400 range^25^. The N400 is a negative deflection in the event related potential (ERP) that peaks approximately 400 milliseconds after word onset, but is often visible from 250 ms onwards. The N400 to a word that is not predicted or is unexpected will have a higher amplitude than if the word was predicted or expected^26,27^. This modulation comes later than the viseme effect.

Are the abilities to use visual and sentential information two sides of the same coin, that is, do they reflect the same predictive process? Before we attempt to answer this question, it is important to be clear what we mean by “prediction”. Different mechanisms that could support prediction have been proposed, including those based on pre-activation^28^, on Bayesian principles^29,30^, on generative models^22,31^, and on predictive coding^32–34^. Given these varying theoretical perspectives, there are different views of what “prediction” is. For example, if prediction is based on pre-activation, anticipatory effects (e.g. evidence that the listener is actively considering a word as a perceptual hypothesis before any acoustic evidence for that word has been heard) can be taken as the litmus test of predictive processing. From a Bayesian perspective, however, processing that is not strictly anticipatory (i.e., where prior knowledge modulates processing of a word as it is being heard) is still predictive^30^. There is also debate about whether prediction is the sole process underlying language comprehension, or whether it is only one of multiple processes^35,36^. Relatedly, there is current discussion about whether a key demonstration of prediction replicates^28,36,37^. In spite of these ongoing debates, there is consensus that, at least under some circumstances (e.g. when the previous context is highly constraining), comprehenders can use contextual information to anticipate upcoming words and their associated features^17,38^. This anticipatory ability is what we mean here by “prediction”.

Can visual and sentential cues therefore be used jointly to predict acoustic-phonetic aspects of spoken words? At first glance, the electrophysiological data might suggest that prediction based on visual information is distinct from that based on sentential information: the N1 is several hundred milliseconds earlier than the N400. The timing of the N400 is such that N400 effects based on sentence context may not even reflect prediction: they are late enough to reflect instead effects of integration (where the current word is being integrated into the ongoing interpretation of the sentence)^39^. If, however, visual and sentential constraints influence the same anticipatory process, we should expect to see effects of sentential context in the same time window as the N1. This hypothesis is supported by other evidence indicating anticipatory effects of sentential constraints on speech comprehension. In visual-world eye-tracking studies, for example, verb-based constraints about the type of noun that is likely to be the grammatical object of the verb can influence processing before the acoustic onset of the noun^17^. Since sentential constraints can thus be used anticipatorily, and predictive processes based on visual information can be detected as changes to the N1, it ought to be possible to observe a modulation of the N1 visual effect by semantic constraints.

To test this potential early modulation, we used magnetoencephalography (MEG) to look at auditory N1 latency and amplitude in an audiovisual speech paradigm. The final words of spoken sentences could be contextually constrained or unconstrained and they began with salient or non-salient visemes. We predicted that the viseme effect (more N1 suppression to salient visemes) would depend on whether the sentence was constrained or not. Such a demonstration would suggest that sentential constraints are being used to predict (i.e., to anticipate) rather than to influence semantic integration. This is because the N1 is not considered to reveal semantic integration and because modulation of the N1 by viseme salience is a signature of form-based processing (i.e., it reflects predictions about the sentence-final word’s initial consonant, not its meaning). Such an interaction would thus suggest that effects of visual and sentential information in audiovisual speech comprehension are indeed two sides of the same coin, that is, that they both reflect the ability of the listener to predict upcoming words. We expected an interaction between viseme salience and sentential constraint to arise in the left hemisphere. We did not have a specific hypothesis about the shape of the interaction. One possibility, for example, was that the viseme effect would be present only in the absence of sentential constraints, as might be expected if the sentential constraints were powerful enough to fully predict the phonological form of the upcoming word and hence to make the visemes uninformative. As noted above, however, the prior literature does not provide clear evidence that word forms are predicted based on sentential context (i.e., N400 effects reflect processes higher than the word-form level). Furthermore, even if word-form predictions are made, it is unclear whether they would be sufficiently unambiguous to neutralize all effects of visual information. Such a specific hypothesis was therefore not warranted. Similarly, other detailed hypotheses about the form of the interaction (e.g., whether sentence and viseme would jointly suppress the neural response and shorten N1 latency) were also not made. We also included the right hemisphere, where we did not expect to find an interaction, as a control.

## Materials and Methods

### Participants

The criteria for participation in the study were as follows: Dutch native speaker, 18–30 years of age, no dyslexia or other language-related or neurological problems, right-handed, normal hearing, normal or corrected-to-normal eyesight and no metal in the body. We determined eligibility based on participants’ self-report. Twenty-five subjects took part in the experiment (6 male, mean age = 22.4 years, SD = 2.25). All signed an informed consent form in accordance with the Declaration of Helsinki. The study was approved by the local ethics committee (CMO region Arnhem/Nijmegen), and participants were paid for their participation. One participant was excluded due to excessive artifacts caused by makeup residue that had been magnetized during a magnetic resonance imaging (MRI) session earlier that day. All were right-handed, with normal hearing and normal or corrected-to-normal vision.

### Experimental materials, design and procedure

**Figure 1:**
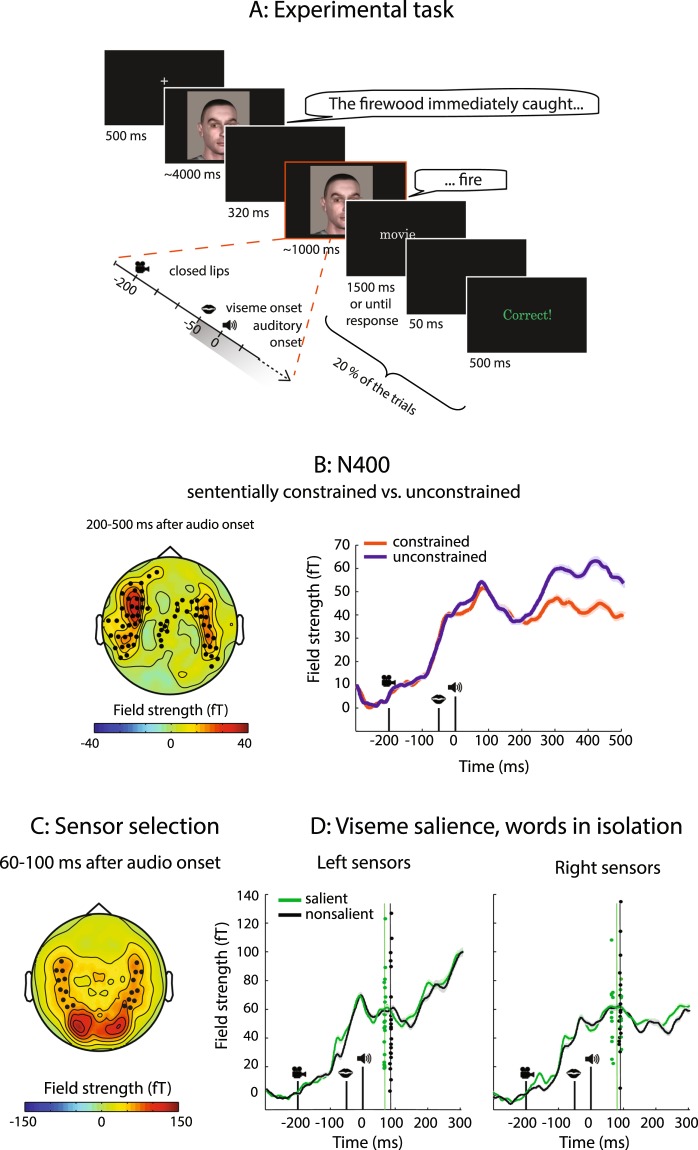
Experimental task, sensor selection, N400 and latency results. (**A**) Trial sequence. A video displays a speaker uttering a Dutch sentence for about four seconds. The final word of the sentence appears after a short blank screen (320 ms). In this example, the auditory contents of the sentence-final target word are made more predictable due to the salient viseme (/f/), and the constraining sentential context. The target word here (‘fire’) is followed by a written word, presented on 20% of the trials. Participants pressed a button to indicate whether it was the same as the previous word. (**B**) N400-effect of sentence context. Left: topography of the difference in activity to words preceded by a sententially unconstrained vs. constrained context, with significant sensors highlighted. Sententially unconstrained words led to stronger neural activity over temporal, parietal and frontal sensors. Right: event-related fields to sententially unconstrained (purple) and constrained sentences (orange), averaged over sensors highlighted on the left. (**C**) Sensors of interest with average N1 topography to videos of single words. The most active left and right temporal sensors are highlighted. (**D**) Event-related fields to words beginning with salient (green line) and non-salient visemes (black line), plotted separately for sensors in the left and right hemisphere. Jackknifed auditory N1 peak latencies for each subject are represented by dots, their means by the vertical lines. In the left sensors, the auditory N1 peaked earlier if the viseme was salient than if it was not.

Participants watched videos of a speaking person who was recorded from the shoulders up (see Fig. 1A). The videos contained spoken sentences. The last word (target word) of the sentence either started with a salient or a non-salient viseme. A salient viseme allowed the upcoming auditory content of the target word to be predictable (relative to a non-salient viseme). The content of the target words could also be predictable depending on the form and meaning of the preceding sentence. We therefore orthogonally manipulated the viseme salience of the initial sounds of sentence-final words and the sentential constraints on those words (see Table 1). This resulted in four experimental conditions: constraining sentential context with salient viseme, constraining sentential context with non-salient viseme, unconstraining sentential context with salient viseme, and unconstraining sentential context with non-salient viseme. Additionally, we presented the spoken target words in isolation to test for the early N1 effect of viseme salience without a sentence context. Here, our manipulation resulted in two experimental conditions: salient viseme and non-salient viseme.

**Table 1:** Target words. IPA = International Phonetic Alphabet.

Target words	English translation	IPA	Frequency	Acoustic word duration (ms)	Visual word duration (ms)	Total clip duration (ms)
fik	fire	[fik]	6.49	293	440	1000
film	film	[film]	174.28	372	720	1160
filter	filter	[ˈfiltər]	2.04	541	640	1040
fit	in good shape	[fit]	5.37	373	600	1040
gif	poison	[xif]	13.56	400	960	1320
gil	scream/shriek	[xɪl]	9.99	330	640	1040
gisteren	yesterday	[ˈxistərə]	131.79	513	640	1000
gips	plaster/gypsum	[xips]	2.36	492	680	1040

Frequency did not differ significantly between /f/- and /x/-words.

#### Target words

We manipulated viseme salience while keeping acoustic features constant across and within conditions. In order to achieve this, we selected two Dutch speech sounds that are phonetically similar but that differ in visual salience: the unvoiced labiodental fricative /f/ (the same sound as an English f) and the unvoiced velar fricative /x/ (similar to the final sound in *‘Bach’*). Fricatives are consonants we produce by forcing air through a narrow constriction. In the case of an /f/, this constriction is between the upper teeth and lower lip, making it visually salient. For /x/, the constriction is created by positioning the back of the tongue close to the soft palate, resulting in a less salient viseme. Based on auditory information alone, Dutch participants can correctly distinguish /f/ and /x/ after having heard approximately equal portions of the sounds^40^. In addition, these two sounds are often confused with each other early in the auditory recognition process^40^. They are also similar in duration. To confirm this, we measured the frication duration for 7/f/ and 7/x/words, yielding means of 143 ms and 147 ms respectively. Since /f/ and /x/ are acoustically similar, additional visual information could make their discrimination easier.

We then chose four words starting with each of these two speech sounds followed by the vowel /ɪ/ to further ensure acoustic similarity. This resulted in eight target words (Table 1). The mean frequencies of the words in the two conditions were not significantly different (t(6) = 0.145, p = 0.89) based on the word frequencies in the SUBTLEX-NL database^41^. We visually inspected the sound clips in Praat^42^ to determine acoustic duration. We determined visual duration (onset to offset of lip-movements) by visual inspection of the video clips in Adobe Premiere Pro 6. To minimize the effect of word repetition and strategic processing related to the onset sounds, we additionally introduced trials ending with 16 filler words, none of which started with /f/ or /x/. The filler words were semantically related but acoustically distinct from the target words (e.g., “verband” (bandage) and “klei” (clay) for the target word “gips” (plaster)). We did not analyse neural activity related to these filler trials.

#### Sentences

We initially constructed 24 different sentences ending with each of the eight target words, which makes a total of 192 sentences. Half of the sentences strongly predicted a particular sentence-final target word, whereas the other half were not strongly constraining, and thus poor predictors of the target words. We pilot tested how predictive the sentences were (without the final words) on a group of 40 Dutch participants (native speakers with no dyslexia) with one of four versions of a pen-and-paper sentence completion test. Participants were asked to indicate which word best completed each sentence. Based on the results of this test, we then selected the 10 most and 10 least reliably predictive sentences for each target word, which gave us a final set of 160 target sentences (see Supplementary Materials for all sentences and Table 2 for examples). The predictive power of the sentences selected for the experiment was on average 80% predictability of the sentence-final word. We also constructed 10 sentences for each of the sentence-final filler words, half of which were predictive and half of which were not.

**Table 2:** The four experimental conditions with example sentences.

	Salient viseme	Non-salient viseme
Sententially constrained	Het brandhout vloog meteen in de FIK *The firewood immediately caught FIRE*	De tanden van een cobra bevatten dodelijk GIF *The teeth of a cobra contain deadly POISON*
Sententially unconstrained	Toen Roel thuiskwam stonden zijn spullen in de FIK *When Roel came home his stuff was on FIRE*	De biologiestudenten lezen een artikel over GIF *The biology students read an article about POISON*

#### Video recording and editing

A male native Dutch speaker with a neutral dialect was chosen as speaker. He was seated in a chair facing the camera. A soft box lighting device was behind the camera to ensure maximal visibility of his facial movements. The speaker was instructed to speak clearly and at a natural pace, but to include a pause before the sentence-final word. Later, a single target word video was chosen and presented at the end of all 20 sentences associated with that word. The recording was done in a soundproof room using a digital HD video camera (JVC HD GY-HM100E) at 1920 × 1080 resolution and 25 progressive frames per second. Sound was recorded with the camera microphone at a sampling rate of 48 kHz.

First, video clips of each sentence and each sentence-final word were selected and cut out from the raw recordings in Adobe Premiere Pro 6. Every sentence and word video started and ended with a neutral face and a closed mouth. The video clips of the lead-in sentences had six frames (240 ms) of neutral face at the beginning and three frames (120 ms) at the end, whereas the word video clips started with three (120 ms) and ended with six frames (240 ms) of neutral face. This enabled us to control the visual gap between lead-in sentence and target word. The sound was noise-reduced in Audacity and its amplitude was root mean square equalized in Praat. We put the edited sound clips back into the video clips in Adobe Premiere Pro 6 without any audiovisual asynchrony or realignment, after which we exported the edited video clips in uncompressed AVI format with a 720 × 480 frame size. Finally, we compressed and exported the video clips as AVI files in the Indeo 5.10 codec using VirtualDub.

#### Stimulus presentation

Each trial started with a fixation cross placed approximately between the eyes of the speaker in the upcoming video. After 1500 ms, the fixation cross was replaced by the lead-in sentence video (in sentence trials) or target-word video (in word trials). In the sentence trials there was a gap during which a uniformly black screen was presented for 320 ms between the lead-in sentence and the sentence-final target word (Fig. 1A). This ensured a constant interval (560 ms) between visual lead-in sentence offset and target word onset. Additionally, it allowed us to use identical target word videos across trials. Importantly, the onset of the auditory content in the target word videos naturally lagged the viseme onset by 50 ms. This lag was constant within and across conditions.

We used two types of trials in the experiment: sentence trials and world trials. The sentence trials made up the first part of the experiment, and the word trials came last. The word trials contained exactly the same target words as the sentence trials, but no fillers. The order of stimulus presentation was pseudorandom throughout the experiment. During sentence blocks, a target word was never repeated on two consecutive trials, and the proportion of fillers and targets was balanced across blocks. In word-only trials the target and filler words were presented in a pseudorandom order.

### Task

While participants performed the task, we recorded their ongoing neural activity with MEG. The video was projected on a screen at a distance of 70 cm from the participant, and the sound delivered binaurally through MEG-compatible air tubes at a comfortable sound pressure level. Stimulus presentation was controlled by a PC running Presentation software (Neurobehavioral Systems). The experiment contained 8 sentence blocks of 40 trials, leading to a total of 160 filler trials and 160 target trials. After the sentence blocks, there was one word-only block with 20 trials per target word, again leading to a total of 160 trials. The inter-trial interval varied randomly between one and two seconds throughout the experiment. All participants saw the same set of stimuli, but the order of presentation differed per participant.

To ensure that participants would pay attention to the target words, we added a word discrimination task on 20% of the sentence trials and 35% of the word trials. During task trials, a written word appeared on the screen immediately after the word video, and the participants had to indicate with a button press whether this word was the same or different from the word they had just heard. As the word discrimination task came later in the epoch than the time of interest for us (that associated with target word processing), these trials were included in the analyses.

Before the experiment began, participants tried two practice trials. During the experiment, there was a break of at least 30 seconds after every block of 40 sentence trials, and a break of at least 60 seconds before the word-only block. After these obligatory breaks were over, participants could press a button to proceed with the experiment whenever they wanted.

#### MEG acquisition

We recorded brain activity using a whole-head MEG (275 axial gradiometers, VSM/CTF Systems) at a sampling rate of 1200 Hz in a magnetically shielded room. Participants’ head position was monitored during the experiment using coils placed at the nasion and in both ear canals, and was corrected during breaks if needed. Horizontal and vertical electro-oculogram (EOG) was recorded using 10-mm-diameter Ag–AgCl surface electrodes. We later used the vertical EOG to aid offline rejection of blink artifacts.

### MEG data analysis

The MEG data were preprocessed and analysed in Matlab (MathWorks, Natick) using the FieldTrip toolbox^43^. We calculated event-related fields (ERFs) time-locked to the onset of the target word videos, and used these to analyse auditory N1 latencies as well as early and late amplitude effects as a function of our experimental manipulations.

#### Calculation of event-related fields

We extracted trials of 1300 ms from the data starting 300 ms before the onset of the target word videos. Trials containing jumps in the MEG signal caused by the SQUID electronics were rejected based on visual inspection. Trials containing excessive muscle artifacts were then rejected after visual inspection of the amount of variance in an epoch with the signal bandpassed at 110–140 Hz. Next, we used independent component analysis^44^ to remove variance in the signal pertaining to eye blinks^45^. Finally, we discarded any remaining trials where the ERF amplitude was more than 4 standard deviations above or below the mean. In total, we rejected an average of 12.75 (SD = 4.80) trials per participant.

Before calculating ERFs for each condition of interest, we low-pass filtered the data at 40 Hz and baseline corrected relative to a 100 ms time window before the onset of the word videos. Finally, we calculated planar gradient transformed ERFs^46^, a procedure which simplifies the interpretation of the sensor-level data because it places the maximal signal above its source^47^. Importantly, this operation removes the polarity of ERF components, making the strength of their deflections from zero across conditions the main information of interest. The error bars in all the ERF figures represent within-subject standard errors^48^.

#### Sensors of interest

We constrained our analyses to temporal sensors, with the exclusion of sensors bordering occipital areas (in order to avoid excessive contamination by visual activity). To further select the sensors of interest, we used the grand averaged ERF data from words presented in isolation. We selected the 10 most active temporal sensors in each hemisphere in a time window corresponding to the auditory N1 component, 60–100 ms after auditory onset (Fig. 1C). We have used the same sensor selection procedure for all previous auditory studies in our lab.

#### Analysis of auditory N1 peak latency

We calculated auditory N1 peak latencies for the target words, separately for each hemisphere. We used a jackknife approach, which allows for robust estimation of latency differences, to estimate the N1 peak latencies^49,50^. Instead of computing one average N1 latency value per participant, we computed as many averages as there are participants while leaving one participant out each time. If the latency is consistent over participants, then the average value will not change substantially depending on which participant is left out. To test whether the conditions of interest exhibited latency differences, we compared the estimated peak latencies for salient vs. non-salient visemes in the time window between 60 and 100 ms after auditory onset, with the t-values corrected to t_corrected_ = t/(n − 1) in order to reduce the false positive error rate^51^. We first compared latencies in the word-only condition, then for words in sentence contexts. To assess whether viseme salience modulations of N1 latency depend on sentential predictability, we also tested the viseme/context interaction by comparing the difference of the viseme effects in the two sentential conditions against each other.

#### Analysis of auditory N1 amplitude

We tested for auditory N1 amplitude differences as a function of the preceding viseme and sentential information. We used nonparametric cluster-based permutation t-tests for paired samples^52^. All reported amplitude p-values are cluster p-values. Cluster-based permutation tests are well suited to test for differences between conditions in time-series data. The test controls for the false alarm rate by taking advantage of the fact that effects are typically clustered in time. We tested for clusters of amplitude differences across samples of a 300 ms long time-window starting from 100 ms before auditory onset (i.e., 50 ms before viseme onset, 100–400 ms after target word video onset,), using 5000 permutations for the generation of the null distribution. We first compared salient to non-salient visemes in the word-only condition, followed by the same comparison for words in a sentence context. We also compared the effect of constraining vs. unconstraining sentential contexts. We also tested the viseme/context interaction. In order to test the viseme x sentential constraint interaction using FieldTrip, we calculated the difference between the two viseme conditions in our sensors of interest, and then compared this difference in a t-test between constraining and unconstraining sentence contexts. Finally, to perform a three-way interaction (viseme x sentential constraint x hemisphere), we calculated the difference of differences of these two conditions in each hemisphere separately (i.e., weak minus strong viseme in unconstrained sentences minus weak minus strong viseme in constrained sentences) and averaged over left sensors in the left hemisphere and over right sensors in the right hemisphere. We then compared these two sets of numbers in a final t-test. It is important to note that we could have, in principle, approached the interaction from an opposite angle, by testing for a difference in the context effect across the two different levels of visual salience. The decision to set up our interaction test the other way was theoretical, as we were primarily interested in how viseme salience works with real words in the context of sentence predictability. Previous research on viseme predictability tended to use syllables without meaning^8^, and our aim was to build from there: first, to show whether the meaning in real words diminishes this early auditory effect, and second, to see whether the meaning conveyed by the sentence context would modulate it further.

#### N400 analysis

To verify that our sentence context manipulation resulted in a classical N400 effect^26^, we used a cluster test to compare ERFs of the sententially constrained and unconstrained sentence-final words. This test searches for groups of sensors that show a significant difference between the two conditions. We collapsed over all time points from 200 ms to 500 ms after auditory onset for this analysis.

### Data sharing

The raw datasets collected during this study as well as the analysis scripts are available for download from the Donders online repository. https://data.donders.ru.nl/collections/di/dccn/DSC_3018012.05_367?5.

## Results

### Task accuracy

On 20% of the sentence trials, participants performed the word discrimination task. They did so with high accuracy (94.63% + 0.52%, mean + SD) suggesting that they paid attention to the target words. In the word-only trials, accuracy was very similar (93.08% + 0.83%).

### N400 amplitude decreases in constrained sentence contexts

We first examined whether our manipulation of sentence context resulted in an N400 effect. The N400 component to a sentence-final word is known to decrease in amplitude in the presence of a constraining context, that is, when the sentence context renders it predictable^26^. This is what we found as well: a large number of sensors showed an attenuated response in the constraining sentential context compared to the unconstraining sentential context (p = 0.015, pre-defined time window of 200–500 ms after auditory onset). The difference topography in this time window (Fig. 1B) suggests that the effect of sentential constraints was present in both hemispheres, but was more pronounced on the left side. It originated from a difference in activity in temporal sensors, as well as a smaller number of parietal and frontal sensors.

### Salient visemes shorten auditory N1 latency in the left hemisphere

We assessed whether viseme salience influenced the peak latency of the auditory N1 to words presented in isolation, outside of a sentence context (Fig. 1D). We were able to extract a reliable N1 peak latency for both viseme conditions (salient and non-salient) in the left hemisphere. In contrast, in the right hemisphere, the jackknife-estimated peak latencies following salient visemes formed a bimodal distribution, indicating two separate peaks of similar amplitude (i.e. an absence of a reliable N1 peak). We therefore compared the peak latencies only in the left hemisphere. There we found that words beginning with salient visemes were associated with an earlier auditory N1 peak than words beginning with non-salient visemes (69 vs. 88 ms, SD 1.15 vs. 1.12, t(23) = 2.25, p = 0.034).

**Figure 2:**
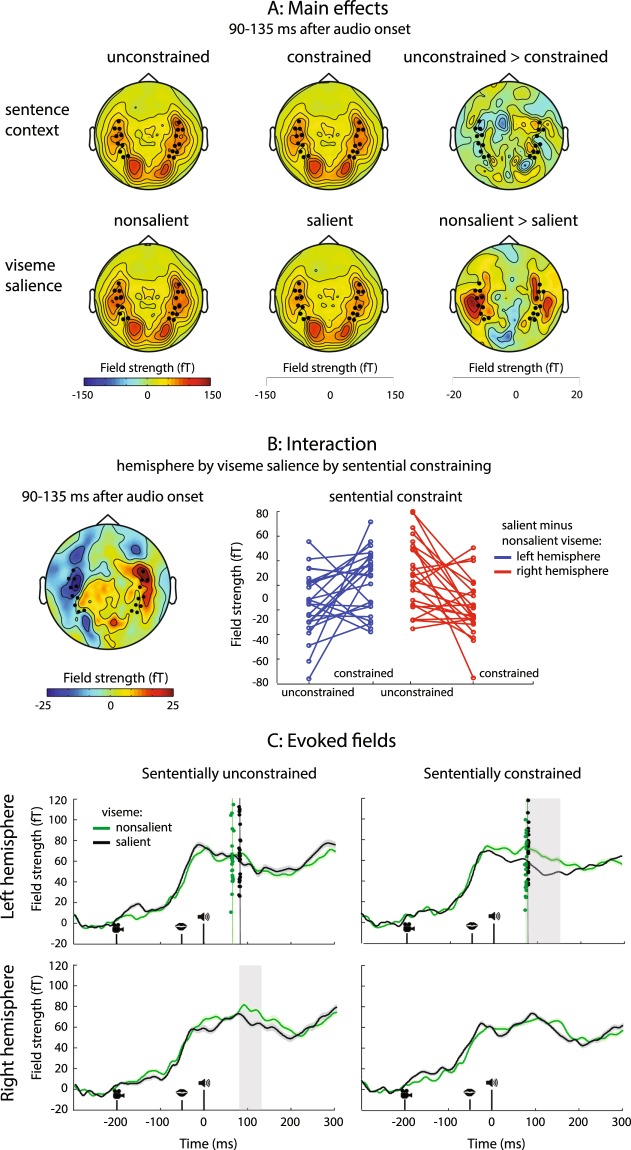
Main effects and interactions on ERF amplitudes. (**A**) Topography of the main effects of sentential constraints and viseme salience in the time window of the significant three-way interaction between hemisphere, viseme salience and sentence context. Sensors of interest are highlighted. (**B**) Left - Topography of the interaction. Right - Individual subject representation of the interaction, in two most prominent sensors on each side. Each red and blue dot represents the difference in the signal between non-salient and salient visemes, under different conditions of sentential constraint. (**C**) ERFs for the salient and non-salient visemes in the two hemispheres under different conditions of sentential constraint. Clusters of significant differences are highlighted. Dots in the upper plots (left hemisphere) represent individual jackknife-estimated N1 latencies, with their means represented as vertical lines.

We next tested whether the viseme effect - earlier N1 peaks to words containing salient compared to non-salient visemes - would continue to be present when the words were embedded in a (constraining or unconstraining) sentence context (Fig. 2C). We performed this comparison in the left hemisphere only. We found that salient visemes shortened the N1 latency in an unconstrained sentential context, in other words when the upcoming word could not be predicted (64 vs. 85 ms, SD 1.8 vs. 1.1, t (23) = 2.15, p = 0.02). We did not find an effect of viseme salience in the constrained sentential context (72 vs. 78 ms, SD 1.13 vs. 0.59, t (23) = 0.98, p = 0.41). However, we did not find evidence for an interaction effect between viseme salience and sentential context (no difference in the viseme effect in constraining compared to unconstraining sentences, mean difference of differences = 15 ms, t (23) = 1.49, p = 0.147). In sum, although the viseme effect persisted when words were embedded in unconstraining sentences, the lack of an interaction effect makes it difficult to estimate the extent to which sentential constraints do or do not influence how much viseme salience influences auditory N1 peak latency.

### The early joint influence of viseme salience and sentential constraints is hemisphere-dependent

We looked at ERFs (up to 300 ms after word onset) to assess whether viseme salience, which exerted a consistent influence on N1 latency in the left (but not right) hemisphere, would also exert an effect on signal amplitude. When words were presented in isolation neither the left (p = 0.137) nor the right (p = 0.131) hemisphere showed evidence of an amplitude difference in early auditory processing following visemes of different salience (Fig. 1C).

We then asked whether viseme salience affects signal amplitude to words presented in the context of a sentence. We found that viseme salience and sentential constraints came together to exert a joint influence on auditory processing, but that the pattern of their interaction depended on the hemisphere (Fig. 2B,C). In both hemispheres, non-salient visemes led to larger auditory responses in the N1 time window. However, this viseme effect was modulated by sentential constraints in opposite ways in the two hemispheres. A viseme effect was evident only in the *presence* of sentential constraints in the left hemisphere, and only in the *absence* of sentential constraints in the right hemisphere (three-way interaction between hemisphere, sentential constraints and viseme salience: p = 0.012, 90–135 ms). In other words, the left auditory cortices responded less to salient visemes when the upcoming word could be predicted based on sentential constraints while the right auditory cortices responded less to salient visemes when the upcoming word could *not* be predicted using sentential constraints (Fig. 2B, left).

Next, we looked at the effect of viseme salience and sentence context for the two hemispheres separately. In the left hemisphere, we found no evidence for an effect of sentential constraints on early auditory responses (main effect of sentential constraints: no clusters found). Viseme salience, in contrast, did exert an effect on the early neural response (main effect of viseme salience: p = 0.012, 80–126 ms after sound onset). The stronger amplitude to non-salient than salient visemes depended on whether a sentence was sententially constraining or not (marginal interaction between viseme salience and sentence context: p = 0.061, 131–159 ms). Namely, a viseme effect was present if the sentences were sententially constrained (p = 0.04, 71–152 ms), but we found no evidence of it if the sentences were sententially unconstrained (p = 0.265).

In the right hemisphere, we observed a reverse pattern of results. We found no evidence of an effect of viseme salience on neural activity (no main effect of viseme: p = 0.196). Conversely, we observed stronger activity to sentence-final words preceded by an unconstraining sentential context relative to a constraining one (main effect of sentential constraint: p = 0.020, 12–51 ms). As in the left hemisphere, in the right hemisphere we also found a combined effect of whether a viseme was salient and whether the sentence was sententially constrained (interaction between viseme salience and sentential constraint: p = 0.044, 87–117 ms). Here, words starting with non-salient visemes led to more neural activity than words starting with salient visemes in sententially unconstraining sentences (p = 0.040, 90–121 ms), but we found no evidence of a viseme effect in sententially constraining ones (p = 0.278).

### No evidence that sentential constraint modulates neural activity before word onset

A potential limiting factor of this study is that the sentence-final words were preceded by a blank screen of 320 ms. During this time window, predictions related to sentential constraint might already begin to influence neural activity. If that were the case, then the ERFs following word onset would partly reflect this already accumulated prediction. This type of prediction accumulation, in turn, might not be an accurate representation of language processing that unfolds at a natural speed.

In order to assess the influence on sentential constraints on the period just before the final word onset, we compared ERFs during this time window. Our epochs here were limited to the blank 320 ms period, starting with a 100 ms baseline just before the blank screen and ending at target word onset. We did not find evidence of a difference between sententially constrained and unconstrained sentences (p = 0.35), indicating that neural differences began only after target word onset. We additionally repeated this test separately in left and right sensors, but found no difference in either (p = 0.32 and p = 0.55, respectively).

## Discussion

In this study, we examined the independent and joint effects of viseme salience and sentential constraints on early auditory processing in sentence-final words. In electrophysiological studies, viseme salience typically affects N1 latency and/or amplitude^5^, while sentential constraints typically affect the N400^25–27,53^. If, however, both effects reflect an increase in the predictability of the upcoming auditory input, and since studies using other paradigms (e.g. eye tracking)^17^, have demonstrated effects of sentential context even earlier than the N1, we predicted that sentential constraints could modulate the early viseme effect by making the viseme more or less predictable. We found that sentential context can indeed have an effect on early language processing (~90 ms after word onset) through modulating reliance on early visual cues, but that the pattern of this effect depended on the brain hemisphere: the left hemisphere integrated probabilistic cues from visual and sentential information, while the right hemisphere gave visual cues priority only when no strong sentential constraints were present.

Before we go on to discuss these results in greater detail, it is important to note where our study stands in terms of ecological validity. The majority of studies on viseme salience investigate the effect in isolated phonemes or syllables^5,7,8,15^. In contrast, we employed a paradigm where full sentences were spoken. However, we introduced a break between the penultimate word and the target word. This manipulation allowed for better experimental control and a higher signal to noise ratio, but it comes at the cost of reducing predictability based on natural prosody and coarticulation. While we believe that our results allow for the conclusion that comprehenders *can* use both viseme salience and sentential constraints to predict upcoming words, they do not necessarily imply that people always do so in natural speech situations. In addition, we repeated the target words throughout the experiment. This might have increased anticipation of their identity in all experimental conditions, which could have led to an attenuated neural signal based on both higher predictability and repetition suppression, and therefore smaller observed differences between the conditions. As the target words were task-relevant, the increased predictability might have also increased attention to them. It remains an open question whether the pattern of observed results would hold with task-irrelevant words. Another caveat we need to make is the following. We have assumed that viseme salience is the only driver of our viseme effect, as previous studies have found the degree of salience relates to the degree of N1 suppression^15^. And indeed, the effect we find goes in the same direction, with more salience meaning more suppression. However, we cannot rule out the possibility that the auditory processing of the /f/ and /x/ sounds we used might have different cortical sources^54,55^. The differences we observed in N1 amplitude may therefore reflect auditory rather than visual differences between the two critical speech segments.

We first replicated a typical N400 effect (a decreased amplitude to words embedded in a constrained compared to an unconstrained sentence context, Fig. 1B). This suggests that participants were anticipating upcoming word forms when the sentential context was constrained, although it might equally reflect ease of integration^39^. We also replicated a viseme effect (shorter N1 latencies to words beginning with a salient viseme compared to a non-salient one) in left temporal sensors, when words were presented outside the context of a sentence, but we did not find a visual salience effect on the N1 amplitude. The latency shortening implies that participants used the visual information from the lip movements to predict upcoming auditory input. This type of latency shortening has been argued to reflect faster auditory processing^7^, and to be insensitive to whether or not the speech sound matches the viseme^15^. One suggested mechanism underlying visual facilitation of auditory speech processing is phase-resetting of activity in auditory cortex due to input from visual motion areas^15,56^.

Crucially, when we investigated auditory activity to words embedded in the context of a sentence, we found that the level of sentential constraints modulated both the N1 latency and its amplitude, through changes in the effect of viseme salience. Both hemispheres exhibited joint sensitivity to viseme salience and sentential constraints, but, interestingly, the effect of these two factors on early auditory word processing were differently expressed. In the left hemisphere, we observed N1 peak latency shortening to salient visemes in the absence of sentential constraints, as well as an amplitude reduction in the presence of sentential constraints. In the right hemisphere, we did not detect reliable N1 peaks (and so did not estimate latencies or a possible hemispheric interaction), but a lower amplitude to salient visemes was evident in the absence of sentential constraints.

A number of studies have demonstrated a suppressed and earlier N1 for words beginning with more salient visemes^5–7^, and one study suggested that the degree of salience could be predictive of the degree of suppression of the BOLD response^8^. We replicated this stronger suppression to words beginning with more salient visemes in the left hemisphere when prior sentence context was constrained, but not when it was unconstrained. In contrast, the N1 *latency* to salient visemes in the left hemisphere was shorter only in the absence of sentential constraints. It is important to note that, even though we found an N1 peak latency shift to salient visemes when the target words were not sententially constrained, and no viseme effect when they were, we did not find evidence of a viseme-context interaction in this analysis. This is partially in line with a recent EEG study that combined sentential constraints with viseme salience, where there was an N1 latency shift only for salient (vs. non-salient) visemes, but no early interaction with sentential constraints^16^.

We also looked at the effect of viseme salience and sentence context on the ERF amplitudes. Both hemispheres were sensitive to a combination of sentential constraints and viseme salience in the N1 time window, but surprisingly, the reliance on viseme salience as a function of sentential constraints differed per hemisphere, with the left hemisphere integrating both effects, but the right hemisphere giving priority to visual cues only in the absence of sentential constraints. In the right hemisphere, we found a higher amplitude for non-salient visemes in the absence of sentential constraints, indicating a reliance on visual cues only when there was no helpful sentential context. In the left hemisphere, conversely, the combined effect of sentential and visual cues suppressed neural activity jointly. Here it appears that the presence of sentential context rendered the word form predictable, and hence also the viseme itself, thus facilitating subsequent auditory processing. The resulting topography of this early hemisphere by context by salience interaction is bilateral and symmetric (Fig. 2B, left).

Why would the joint effect of viseme salience and sentence context (both of which make an upcoming word more predictable) lead to a reduction in the early auditory neural response to words in the left hemisphere? Recent research proposes a major role for stimulus likelihood. Namely, expecting an upcoming auditory stimulus attenuates the auditory N1 both for pure tones^14,57–59^ and for spoken words^60^. Auditory predictions related specifically to viseme salience also cause this early attenuation^8,15^.

Where in the brain do these predictabilities exert their influence? The topographies of our early effects suggest a broad bilateral effect in the temporal lobes for viseme salience (Fig. 2A), and a slightly more anterior, more constrained effect for the interaction between viseme salience and sentential context (Fig. 2B). Broad activity along the superior temporal sulcus has previously been found both when people observed lip movements without sound and when they heard speech^61^. The sensitivity to mismatch between visual and auditory speech has also been suggested to rely on a feedback signal from the superior temporal sulcus^15^. In addition, the anterior temporal cortex has been implicated in several studies of sentential processing^62,63^, and we find it a likely candidate for the source of our interaction effect. In fact, in one study activity related to the predictability of semantic priming was localized in the left anterior superior temporal gyrus^64^, indicating a sensitivity to probabilistic semantic processing. In other words, our interaction of probabilistic processing appears more closely associated with areas corresponding to semantic processing than to those related to viseme processing alone.

A striking finding in this study is that both cortical hemispheres displayed an early sensitivity to viseme salience and sentential context, but that the pattern of this sensitivity differed. We did not expect to find a hemispheric interaction; however, the topography of the interaction appears convincingly symmetric, suggesting that it is constrained to the same brain area. It is possible that the presence of the viseme effect in the N1 window allowed us to observe predictive processing more closely (through perturbing the predictive system so to speak), and that this is why effects of sentence context on predicting word form became evident only in conjunction with the viseme effect. This finding adds to a growing body of research that demonstrates an organization of language processing within the ventral stream which is bilateral, but with a hemispheric asymmetry in activation^65,66^. Behavioural research also supports the idea that the left and right hemisphere both process visual information, but in slightly different manners. For example, it has been claimed that the right hemisphere relies on surface-type visual information longer, whereas the left hemisphere has quicker access to deeper levels of lexical representation: when participants are asked to recognize a letter in a visual stimulus, the right hemisphere, as opposed to the left hemisphere, displays no word superiority effect^67^. In addition, when people have to complete words based on the first few letters, the right hemisphere displays a stronger effect of priming by previously seen words if the case in which the prime was presented matches the case in which the beginning of the target word is presented^68^. Our observation that right hemisphere processing was sensitive to a main effect of sentence context early on, is in line with these findings, suggesting that the right hemisphere processed the word form in this early time window, whereas the left hemisphere already had access to its meaning. This is also in agreement with behavioural research that suggests that the right hemisphere accesses semantic information later, with semantic priming showing effects with a larger prime-to-target stimulus onset asynchrony than in the left hemisphere^69–74^.

Neural evidence also shows that sampling speed in auditory cortex in the two hemispheres differs, with the left hemisphere dominating in faster gamma sampling^75^, compared to the right hemisphere which is more reliant on slower theta sampling^76^. Here we show that, while *probabilistic* language processing is also bilateral, the pattern of neural responses conforms to the functional specificity of the two hemispheres. In other words, sentential and visual predictions interact in functionally different ways in the two hemispheres. Perhaps due to this difference in sampling speed, it appears that the left hemisphere was capable of combining the two probabilistic computations early on, while the right hemisphere was slower to integrate the viseme into the word context. In the right hemisphere, we found a viseme effect only in the absence of sentential constraints, indicating a stronger reliance on visual information compared to the left hemisphere. The left hemisphere, in contrast, appears able to combine the probabilistic information involved in sentential constraints and viseme salience, whereby the sensitivity to visemes becomes more pronounced in the presence of a semantic context.

## Conclusion

Spoken language processing requires integration of information across time, and one of the means that comprehenders have at their disposal to achieve this is that they can make predictions about upcoming content based on preceding content. These predictions vary in strength, arise from different cues, and are made at different levels of language processing. It remains to be determined what the functional mechanism is (e.g., pre-activation, Bayesian inference, generative modelling or predictive coding), but at the neural level it appears that there is suppression of neural activity to predictable sounds and words. Our study sheds light on the joint effects of viseme salience and sentential constraints. We found that both of these factors have an effect on early auditory processing (in the N1 range). The two hemispheres however handled this combined information differently, with the right hemisphere giving priority to visual information in the absence of strong sentential constraints, and the left hemisphere combining visual and sentential information. This speaks to a complex hierarchy of predictions in language processing, one that is reliant on general probabilistic processing mechanisms but is simultaneously highly dependent on the functional specificity (and lateralization) of the associated cortical areas.
